# Change in college student health and well-being profiles as a function of the COVID-19 pandemic

**DOI:** 10.1371/journal.pone.0267724

**Published:** 2022-05-02

**Authors:** Stephanie T. Lanza, Courtney A. Whetzel, Ashley N. Linden-Carmichael, Craig J. Newschaffer

**Affiliations:** 1 The Edna Bennett Pierce Prevention Research Center, College of Health and Human Development, The Pennsylvania State University, University Park, PA, United States of America; 2 Department of Biobehavioral Health, College of Health and Human Development, The Pennsylvania State University, University Park, PA, United States of America; Wonkwang University, REPUBLIC OF KOREA

## Abstract

**Objective:**

The COVID-19 pandemic has potential for long-lasting effects on college students’ well-being. We examine changes from just before to during the pandemic in indicators of health and well-being and comprehensive profiles of health and well-being, along with links between covariates and profiles during the pandemic.

**Participants:**

1,004 students participated in a longitudinal study that began in November 2019.

**Methods:**

Latent class analysis identified health and well-being profiles at both waves; covariates were included in relation to class membership.

**Results:**

Mental health problems increased, whereas substance use, sexual behavior, physical inactivity, and food insecurity decreased. Six well-being classes were identified at each wave. Baseline class membership, sociodemographic characteristics, living situation, ethnicity, coping strategies, and belongingness were associated with profile membership at follow-up.

**Conclusions:**

COVID-19 has had significant and differential impacts on today’s students; their health and well-being should be considered holistically when understanding and addressing long-term effects of this pandemic.

## Introduction

Prevalence of mental health disorders and rates of substance use peak during young adulthood (approximately ages 18 to 25), with approximately 13.8% of young adults meeting criteria for a past-year major depressive episode, 10.1% for a past-year alcohol use disorder, and 7.6% for a past-year illicit drug use disorder [1]. The Coronavirus disease (COVID-19) pandemic significantly disrupted the lives of many young adults, with unique impacts on those attending college. In an effort to curb the spread of COVID-19, in March 2020 more than 1,300 colleges and universities across the US shifted to online-only instruction and closed on-campus housing, leading many college students to leave their on-campus housing and move home with family [2]. The unexpected shift impacted nearly every aspect of student life, including their living situation, learning environment, grading system, social setting, and economic circumstances. The switch to online learning would be expected to deepen disparities in higher education, as this learning mode has been shown to adversely impact certain groups of less advantaged students more strongly [3]. Some students had insufficient access to reliable technology, which has been linked to lower student achievement [4].

In addition to academic challenges, such sudden, dramatic changes in students’ lives led to much stress and uncertainty among today’s college students [5]. The American College Health Association [6] examined the effects of the pandemic on 14 US college campuses and more than 18,000 students, finding that 66% of students reported increased financial stress, 33% reported a change in living situation, 41% witnessed race-based discrimination, and 60% had difficulty accessing mental health care. Based on prior public health crises, epidemics, and pandemics (e.g., H1N1, Ebola) [7,8]; and emerging evidence of the current pandemic [9], there is great potential for long-lasting effects on substance use and mental health. Consequently, there is an urgent need to study the various markers of health and well-being among today’s college students.

Emerging research has examined the impact of the COVID-19 pandemic on young adult mental health and substance use behavior with a primary focus on modeling pre-post changes in individual risk factors, symptoms, or outcomes. For example, a CDC report found elevated levels of mental health problems and suicidal ideation overall in April-June 2020 relative to April-June 2019, with disproportionate impact on younger adults [10]. Other studies contrasted behavior before versus during the pandemic and found that, while the overall prevalence of substance use among adolescents and adults (i.e., the percentage of individuals reporting any substance use) decreased, among those who used substances, frequency and quantity of certain substances such as cannabis and alcohol increased [11–14]. Examination of simple bivariate associations between the onset of the pandemic and health- and well-being-related outcomes of interest may not be adequate to elucidate more complex inter-relationships affecting quality of life in this population. Considering the intersection between multiple aspects of health and well-being may be revealing, given evidence suggesting that higher levels of anxiety, depression, and stress are associated with increases in alcohol and tobacco use [14–17]. The impact of the COVID-19 pandemic on students’ lives was swift and broad, so considering health and well-being broadly in this population may provide new information on subgroups of students with particular needs.

Latent class analysis (LCA) [18] is an ideal approach for holistic examination of multidimensional patterns of well-being among college students. LCA can simultaneously consider individuals’ substance use and other health behaviors, as well as other indicators of well-being most impacted by the pandemic (e.g., mental health symptoms, physical activity, food insecurity) to divide a population into underlying latent classes—in this case, subgroups of individuals that share common profiles across a set of health and well-being markers. Such an analysis may lead to a more comprehensive understanding of college student wellness. Further, to identify students most at risk and in need of university counseling or other services, it is also critical to identify individual-level characteristics associated with latent class membership. Females and individuals from certain NIH-designated health disparity populations (sexual/gender minorities, socioeconomically disadvantaged groups, racial-ethnic minorities) have consistently been found to be at risk for enduring negative impacts of COVID-19, including decreased emotional well-being, increased mental health problems (anxiety, depression), academic-specific stress, and substance use [9,11,17,19–22]. The transition to remote learning specifically also presented unique challenges for many–particularly sexual/gender minorities and socioeconomically disadvantaged individuals. Many sexual/gender minorities were forced to move back home to an unsafe or unaccepting environment [23,24]. Emergency remote learning also revealed a significant “digital divide” among socioeconomically disadvantaged students [25], with lower-income college students reporting higher levels of anxiety and stress than students from wealthier families [20].

To comprehensively understand changes in college students’ health and well-being in response to COVID-19, the current study used data from a longitudinal study of college student health and well-being to assess multiple aspects of health and well-being among students in November 2019, just before the COVID-19 pandemic, and in May 2020 when students had transitioned to full remote learning. By applying LCA to longitudinal data, we can quantify change over time in the most relevant configurations of student health and well-being, as well as the probability of individuals transitioning in class membership from baseline to follow-up. More specifically, the current study first characterized profiles of college student health and well-being at each wave, using LCA to identify classes of students based on a comprehensive set of 11 indicators of health and well-being assessed over time. Then, class membership at follow-up was examined as a function of baseline class membership, sociodemographic characteristics, coping strategies during the pandemic, and feelings of belonging.

## Materials and methods

### Participants and procedure

In November 2019, college students were recruited from a large, multi-campus public university in the Northeastern region of the U.S. via direct email using a current database of all first- and second-year undergraduate students at the main campus and all undergraduates at selected campuses throughout the state. Of the 32,831 unique individuals contacted by email, *n* = 4,737 (14.4%) provided informed consent and completed an online screener to determine eligibility. Eligibility criteria were (1) between ages 18–24 and (2) current undergraduate enrollment at one of nine university campuses. Of those who provided consent, *n* = 4,302 (90.8%) were determined eligible and immediately completed the survey, which had an average completion time of 10 minutes. An additional 49 eligible students responded to a public link to the survey on the study website; thus, the total sample size for this survey was *n* = 4,351. Individuals who completed the survey were compensated $5 and had a 1/100 chance to win a $100 gift card raffle. Data collection occurred during November and December of 2019. All participants were asked to indicate their willingness to be contacted for future research; 2,557 (58.8%) agreed to be contacted again. Prompted by the emergence of the COVID-19 pandemic and the abrupt transition of the university to fully remote education, a follow-up survey of these 2,557 initial respondents was initiated in May 2020 (again, those completing were compensated $10 plus a 1/100 chance to win a $100 gift card raffle). The 1,004 (39.3%; mean/SD age of 19.3/1.4 years; 61.8% female) students completing this follow-up questionnaire formed the analytic sample for this study; note that follow-up data were collected prior to a COVID-19 vaccine becoming available to all adults in the United States. Also note that beginning March 16, 2020 (immediately after spring break), the University switched the format of all classes to be fully remote for the remainder of the semester. On-campus housing and dining halls remained closed during that time and all students were encouraged to remain at home. All extracurricular activities were cancelled, and faculty and staff were encouraged to telecommute if job functions could be performed remotely.

### Measures

Measures included demographic and descriptive characteristics assessed at baseline, a limited set of measures directly related to personal response to the pandemic collected only on the follow-up survey, and a set of mental health, substance use, and other well-being and health behavior measures collected at both waves. See S1 and S2 Appendices for complete survey instruments used at Waves 1 and 2, respectively. Descriptive statistics appear in Table 1.

**Table 1:** Descriptive statistics and tests of change in individual health and well-being indicators (*n* = 1,004).

**Baseline measures**			
**Measure**			**Proportion or M (SD)**
*M* (*SD*) age			19.3 (1.4)
Sex (% female)			61.8%
Sexual minority status (% SM)			12.9%
*Racial/ethnic group*:			
Hispanic/Latinx			7.3%
NH Black			6.1%
NH Asian			10.6%
NH white			70.9%
Other/multiracial group			5.2%
*Class standing*:			
First or second year			75.5%
Third, fourth, or other year			24.5%
First-generation student			29.9%
**Measures collected only at follow-up**			
**Measure**			**Proportion** **or M (SD)**
Not living with parents			12.5%
Consistent social distancing			41.3%
*Coping strategies*			
Healthy behaviors			66.2%
Connect socially			74.8%
Smoking/vaping more			4.8%
Drinking alcohol			18.2%
Using cannabis			8.0%
Eating high-fat or sugary foods			39.6%
**Repeated measures**			
**Measure**	**Wave 1: before COVID-19 pandemic**	**Wave 2: during COVID-19 pandemic**	**Test of change in mean/rate**
*Latent class indicators of health and well-being*			
Elevated depression symptoms	44.1%	61.2%	χ^2^(1) = 84.5, *p* < .0001
Elevated anxiety symptoms	23.0%	24.9%	χ^2^(1) = 1.8, *p* = .208
Past-month heavy episodic drinking	35.0%	17.9%	χ^2^(1) = 113.6, *p* < .0001
Past-month high-intensity drinking	11.8%	5.6%	χ^2^(1) = 32.2, *p* < .0001
Past-month nicotine vaping	16.4%	9.0%	χ^2^(1) = 56.3, *p* < .0001
Past-month cigarette use	5.3%	2.2%	χ^2^(1) = 18.0, *p* < .0001
Past-month cannabis use	16.2%	11.4%	χ^2^(1) = 22.3, *p* < .0001
Past-month sexual activity	46.8%	25.0%	χ^2^(1) = 166.7, *p* < .0001
No physical activity	26.4%	22.2%	χ^2^(1) = 6.8, *p* = .011
Food insecurity	33.0%	20.5%	χ^2^(1) = 61.8, *p* < .0001
Feelings of not belonging	1.18 (0.99)	1.08 (0.96)	*t*(986) = 3.6, *p* = .0003

NH = Non-Hispanic/Latinx.

#### Measures at baseline

On the initial survey, participants were asked to report their *age*, *sex assigned at birth* (male, female, intersex), *current gender identity* (male, female, gender nonconforming, trans male, trans female, different identity), and *sexual identity* (heterosexual/straight, bisexual, gay, lesbian, queer, none of these options). An indicator of *sexual minority status* was coded 1 if gender was not male or female, or if sexual identity was not reported as heterosexual/straight. Participants reported *race or ethnicity* by selecting all categories that apply (white; Hispanic, Latino/a, or Spanish; Black or African American; Asian; Native American or Alaska Native; Middle Eastern or North African; Native Hawaiian or other Pacific Islander; some other race or ethnicity). For the current study, the following racial/ethnic groups were examined: Hispanic, non-Hispanic (NH) Black, NH Asian, NH white; and other race(s). Students who identified only Black and those who identified as Black and white, but no other race/ethnicity, were combined to form the NH Black group. Participants reported *class standing* (first, second, third, fourth, or other year in school), from which an indicator of upper-class standing was coded as 1 for third, fourth, or other year and 0 for first or second year. An indicator of *first-generation status* was coded 1 if participants reported that neither parent had a Bachelor’s or higher degree.

#### Measures of covariates at follow-up

Because the pandemic and the transition to remote education dramatically impacted student living arrangements, participants were asked, “Who are you living with today?” A binary indicator for *not living with parents* was created, coded 1 if they had not selected the option for parents, caregivers, stepparents, etc. and 0 otherwise. *Consistent social distancing* according to national guidelines was assessed with the measure, “Individuals vary in their ability and interest in practicing social distancing, that is, maintaining at least 6 feet between themselves and others (not including those they live with). Please indicate how often you have practiced social distancing during the past two weeks.” Response options ranged from 1 = not at all to 7 = all of the time; an indicator of consistent social distancing was coded as 1 for all of the time, 0 otherwise. Participants checked any of 11 *coping strategies* that they felt applied in response to the question, “To cope with social distancing and isolation, are you doing any of the following?” Separate indicators of engaging in each of the following coping strategies were considered in the current study: Engaging in healthy behaviors; Making efforts to socially connect with friends; Smoking more cigarettes or vaping more; Drinking alcohol; Using cannabis/marijuana; and Eating high-fat or sugary foods.

#### Repeated measures of health and well-being

We considered ten indicators of health and well-being available at both waves. Depression symptoms were measured using the shortened Center for Epidemiologic Studies Depression Scale (CESD-10) [26–28], a 10-item scale assessing the extent to individuals experienced various feelings during the past week (e.g., I felt lonely; I felt that everything I did was an effort). Items were summed for each individual, with scores ranging from 0 to 29 at Wave 1 (*M* = 10.34; *SD* = 6.21) and from 0 to 30 at Wave 2 (*M* = 13.12; *SD* = 6.93). A binary indicator of *elevated risk for depression* was coded 1 for scores over 10.0 and 0 otherwise [27,28].

Anxiety was measured using the anxiety subscale of the Counseling Center Assessment of Psychological Symptoms-34 (CCAPS-34) [29], a 6-item anxiety scale indicating the extent to which each item described individuals (e.g., my heart races for no reason; my thoughts are racing). Items were summed for each individual, with scores ranging from 0 to 4 (Wave 1: *M* = 1.31, *SD* = 1.03; Wave 2: *M* = 1.34, *SD* = 1.07). As described in Locke et al., elevated risk for anxiety was coded 1 for scores over 2.10 and 0 otherwise.

Participants were asked to report whether they engaged in a number of substance use behaviors, each with the response options of 1 = No, 2 = Yes, but not within the last 30 days, or 3 = Yes, within the last 30 days. A binary indicator for *past-month substance use* was created such that responses of 3 were coded as 1, otherwise 0 for each of the five following behaviors: heavy episodic drinking (i.e., 4+/5+ drinks in one sitting for female/male participants), high-intensity drinking (i.e., 8+/10+ drinks in one sitting for female/male participants), any nicotine vaping, any cigarette use, and any cannabis use.

Participants reported *recent sexual activity* by responding to the question, “Have you been sexually active in the past month?” with responses coded 0 for no, 1 for yes.

An indicator for *no physical activity* was created based on participants’ response about activity for at least 30 minutes, 3 times a week (adapted from Cardinal [30] and Nigg [31]) over the past month; responses were coded 1 for achieving this during no weeks and 0 for some or all weeks.

An indicator of *food insecurity* was created based on responses to two questions about insufficient money for food: “Within the past month, I worried whether my food would run out before I got money to buy more,” and “Within the past month, the food I bought just did not last and I did not have money to get more.” The indicator was coded 1 if either item was “sometimes true” or “often true” and 0 otherwise [32].

#### Repeated measure of feelings of not belonging at the university

A single question asked, “When you think about [university name], how often, if ever, do you wonder: Maybe I don’t belong here?” with response options of 0 = never, 1 = hardly ever, 2 = sometimes, 3 = frequently, and 4 = always.

### Analytic plan

All statistical analyses were conducted in SAS (Version 9.4, Cary, NC). Differences across waves in means on continuous variables were tested with t-tests; differences across waves in proportions for each nominal variable were tested using McNemar’s test.

At each wave, we used LCA to estimate latent classes of college student well-being. SAS PROC LCA (Version 1.3.2) [33] was used to identify underlying subgroups of students based on their broad pattern of health and well-being. Separately for each wave, 10 binary variables indicating aspects of recent health and well-being were included: elevated depression symptoms, elevated anxiety symptoms, heavy episodic drinking, high-intensity drinking, cigarette use, vaping nicotine, cannabis use, sexual activity, no physical activity, and food insecurity. Models with 1 through 7 classes were estimated; identification of each model was examined by comparing the solutions obtained across 1000 sets of random starting values. Model selection was guided by information criteria, as well as model identification, interpretability, and parsimony. Lower Akaike’s information criterion (AIC), Bayesian information criterion (BIC), and sample size adjusted BIC (aBIC) values indicate improved balance between model fit and parsimony, and higher entropy values indicate higher classification certainty.

We then investigated the plausibility of measurement invariance across waves using latent transition analysis (LTA) [18]. Invariant measurement would indicate that all latent classes maintain the same meaning across waves, even though individuals can change class membership. When measurement is invariant, LTA can be used to estimate the probability of individuals transitioning over time in membership among a set of latent classes. If measurement invariance does not hold, class interpretation and even class enumeration may differ across waves. In this case, LTA may be used to estimate the probability of membership in a particular latent class at follow-up conditional on membership in a baseline latent class. LTA models that do not constrain measurement to be equal across time, however, are heavily parameterized and can thus have issues with model identification. Given that this occurred in the current study, we assigned individuals at each wave using modal class assignment (i.e., at a certain wave, individuals are assigned to the class associated with their highest posterior probability of membership) in order to calculate the probability of class membership at follow-up conditional on class membership at baseline.

Finally, covariates were examined in relation to latent class membership at baseline and at follow-up to characterize the composition of each latent class; we report the differences across classes in the proportion or mean level of each covariate. Although all pairwise comparisons can be tested (i.e., the difference in the proportion with a certain characteristic between Class 1 and Class 2, Class 1 and Class 3, and so on), to help control the Type I error rate we relied on a simple omnibus test of the overall association for each covariate.

### Ethics statement

At both waves, all participants indicated their consent to participate in the survey by selecting the option “Yes” in response to the question, “Do you consent to participate in this study?” prior to providing any survey data. Participants consented on their own behalf. All study procedures were reviewed and approved by the university’s Institutional Review Board.

## Results

### Changes in individual indicators of student well-being

Ten indicators of student health and well-being were assessed just before the pandemic, in November 2019, and again during the pandemic, shortly after students were required to leave campus and learn remotely, in May 2020 (see Table 1). The prevalence of students with elevated levels of depression symptoms increased significantly from 44.1% to 61.2% (*χ*^*2*^(1) = 84.5, *p* < .0001), whereas elevated levels of anxiety symptoms were largely unchanged (*χ*^*2*^(1) = 1.8, *p* = .208). The prevalence of each past-month substance use behavior decreased significantly, by roughly half for each alcohol and nicotine use behavior and a smaller decrease in the rate of cannabis use (*p* < .0001 for each). The prevalence of past-month sexual activity dropped by nearly half to 25.0% (*χ*^*2*^(1) = 166.7, *p* < .0001); physical inactivity also dropped slightly to 22.2% (*χ*^*2*^(1) = 6.8, *p* < .011). Likely due to the fact that 87.5% of students reported living with their parents at Wave 2, the rate of food insecurity dropped from 33.0% to 20.5% (*χ*^*2*^(1) = 61.8, *p* < .0001). In sum, when considering each indicator of health and well-being, students fared significantly worse on just one indicator (elevated depressive symptoms), were unchanged on one (elevated anxiety symptoms), and showed improvement on the remaining eight (substance use behaviors, sexual activity, physical inactivity, and food insecurity).

### Student well-being profiles before and during the COVID-19 pandemic

At each wave, models with one through seven latent classes of health and well-being patterns were considered. At *baseline*, the information criteria and model identification suggested models with four through seven profiles (see Table 2); the 6-class model was selected based on class separation and interpretability, and the fact that no classes had fewer than 5% of participants. Parameter estimates for the six-class model at baseline are shown in Table 3, ordered by class prevalence. Class 1 (35.9% of students) was labeled “Healthy, Low-Risk” as this class was unlikely to report any of the 10 indicators of problematic health and well-being. Class 2 (24.8%) was characterized by a very high rate of elevated depression symptoms but low rates of endorsing each of the other indicators of well-being; thus, we labeled this class “Depressed.” Class 3 (15.1%) was defined by elevated rates of both depression and anxiety symptoms, recent sexual activity, and food insecurity, and thus was labeled “Poor Mental Health, Sex, Food Insecure.” Class 4 (10.7%) involved near-unanimous heavy episodic drinking and the highest rate of high-intensity drinking, along with a fairly high likelihood of recent sexual activity; We labeled the class “Partiers (Alcohol, Sex).” Class 5 (8.2%), labeled “Partiers (Polydrug, Sex),” comprised students with higher rates of heavy episodic drinking, vaping, and cannabis use, as well as past-month sexual activity. Finally, Class 6 (5.4%) was labeled “Highest Risk,” as it captured students with elevated rates of nearly all problematic health indicators. Of all classes, these students reported the highest rates of elevated depression and anxiety symptoms, cigarette use, cannabis use, recent sexual activity, and no physical activity; they also had high rates of heavy episodic drinking, nicotine vaping, and food insecurity.

**Table 2:** Summary of information to select latent class model of college student health and well-being at baseline and at follow-up.

Latent class	Identification ^a^	AIC	BIC	aBIC	Entropy
*Baseline assessment*					
1	100%	1549.7	1598.8	1567.0	1.00
2	100%	881.7	984.8	918.1	0.77
3	100%	701.0	858.2	756.5	0.73
4	44%	623.8	**835.0**	698.4	0.72
5	22%	596.4	861.6	**690.1**	0.72
6	19%	579.7	898.9	692.5	0.71
7	8%	**567.3**	940.5	699.1	0.74
*Follow-up assessment*					
1	100%	1100.5	1149.8	1118.0	1.00
2	100%	659.5	762.6	695.9	0.80
3	100%	479.4	**636.5**	534.9	0.71
4	64%	451.0	662.2	**525.6**	0.76
5	19%	435.4	700.6	529.1	0.76
6	36%	**430.5**	749.7	543.2	0.82
7	2%	431.9	805.2	563.8	0.81

*Notes*. AIC = Akaike information criterion; BIC = Bayesian information criterion; aBIC = Sample-size adjusted BIC. At each wave, bold font used to indicate minimum value of each information criterion.

^a^ Refers to the percent of 1000 sets of random starting values that result in parameter estimates corresponding to the maximum-likelihood solution.

**Table 3:** Six-class model of college student health and well-being at baseline.

*Latent class at baseline*:	Healthy, Low-Risk	Depressed	Poor Mental Health, Sex, Food Insecure	Partiers (Alcohol, Sex)	Partiers (Poly-Drug, Sex)	Highest Risk
*Percentage of students*:	35.9%	24.8%	15.1%	10.7%	8.2%	5.4%
*Mental health*						
Elevated depression level	0.014	**0.760**	**0.866**	0.386	0.302	**0.991**
Elevated anxiety level	0.002	0.324	**0.589**	0.159	0.474	**0.755**
*Past-month substance use*						
Heavy episodic drinking	0.178	0.037	0.322	**0.998**	**0.943**	**0.739**
High-intensity drinking	0.000	0.000	0.000	**0.507**	0.474	0.404
Nicotine vaping	0.042	0.008	0.147	0.009	**0.907**	**0.887**
Cigarette use	0.009	0.000	0.023	0.000	0.264	0.436
Cannabis use	0.039	0.002	0.191	0.277	**0.612**	**0.742**
*Other indicators*						
Recent sexual activity	0.402	0.210	**0.799**	**0.514**	**0.720**	**0.721**
No past-month physical activity	0.189	0.379	0.325	0.181	0.112	0.460
Food insecure	0.188	0.305	**0.676**	0.240	0.367	0.**558**

*Notes*. Classes presented in descending order according to class size. Probabilities greater than 0.5 in bold to facilitate interpretation.

Based on the information criteria and model identification at *follow-up*, we considered models with three through six profiles (see Table 2); the 6-class model was selected based on class separation, interpretability, and the unique interpretations of smaller classes that were identified. Parameter estimates for the six-class model at follow-up are presented in Table 4, ordered by class prevalence. Class 1 (42.6% of students) was characterized by a very high rate of elevated depression symptoms but low rates of endorsing each of the other indicators of well-being, similar to Class 2 at baseline; thus, we labeled this class “Depressed.” Class 2 (36.4%) was labeled “Healthy, Low-Risk,” as this class had similar item-response probabilities to Class 1 at baseline. Class 3 (6.7%) was defined by elevated rates of both depression and anxiety symptoms and risky alcohol use, and thus was labeled “Poor Mental Health, Heavy Alcohol Use.” This is the only class at follow-up characterized by high-intensity drinking. Class 4 (6.6%) involved elevated depression symptoms and recent sexual activity, and is the only class characterized by a high rate of cannabis use; thus, we labeled the class “Depressed, Cannabis Use, Sex.” Class 5 (3.8%) was labeled “Partiers (Alcohol, Sex)” as the interpretation is similar to Class 4 at baseline. We note, however, that the rate of high-intensity drinking in this class is somewhat lower at follow-up. Finally, Class 6 (3.8%) was labeled “Poor Mental Health, Food Insecure, Low PA” as it captured students with elevated depression and anxiety symptoms, no physical activity, and food insecurity, as well as moderate rates of recent sexual activity. We note that no physical activity and food insecurity are unique to Class 6.

**Table 4:** Six-class model of college student health and well-being at follow-up.

*Latent class at follow-up*:	Depressed	Healthy, Low-Risk	Poor Mental Health, Heavy Alcohol Use	Depressed, Cannabis Use, Sex	Partiers (Alcohol, Sex)	Poor Mental Health, Food Insecure, Low Physical Activity
*Percentage of students*:	42.6%	36.4%	6.7%	6.6%	3.8%	3.8%
*Mental health*						
Elevated depression level	**0.996**	0.051	**0.998**	**0.832**	0.128	**0.997**
Elevated anxiety level	0.370	0.022	**0.565**	0.355	0.003	**0.588**
*Past-month substance use*						
Heavy episodic drinking	0.056	0.052	**0.996**	0.410	**0.981**	0.006
High-intensity drinking	0.000	0.000	**0.579**	0.000	0.360	0.000
Nicotine vaping	0.015	0.017	0.400	0.481	0.348	0.105
Cigarette use	0.000	0.008	0.094	0.043	0.150	0.109
Cannabis use	0.044	0.029	0.360	**0.610**	0.262	0.195
*Other indicators*						
Recent sexual activity	0.132	0.210	0.475	**0.658**	**0.501**	**0.503**
No past-month physical activity	0.242	0.190	0.169	0.181	0.042	**0.636**
Food insecure	0.198	0.128	0.466	0.064	0.079	**0.974**

*Notes*. Classes presented in descending order according to class size. Probabilities greater than 0.5 in bold to facilitate interpretation.

### Class membership transitions from baseline to follow-up

Despite the fact that we selected a 6-class model at each wave, and the models were estimated from the same sample, the fundamental characteristics of the classes varied considerably across waves. Three classes had similar enough item-response probabilities across waves to merit sharing the same label as classes at baseline: Healthy, Low-Risk; Depressed; Partiers (Alcohol, Sex). Not surprisingly, given the vastly different rates of health and well-being indicators after the start of the pandemic, the nature of the remaining classes were quite different. Indeed, at baseline three classes were characterized by elevated depression levels, whereas at follow-up four classes had this as a defining feature. Similarly, at baseline two classes–the Partiers (Polydrug, Sex) and Highest Risk classes–had very high rates of vaping (0.907 and 0.887, respectively), whereas at follow-up, after the university switched to remote learning and many students moved home, vaping was not a defining characteristic of any class. The considerable differences in class interpretation essentially guarantee that this sensitive test would provide statistical evidence against measurement invariance, although a formal test of measurement invariance across waves was not possible due to issues of model identification when estimating latent class variables at both waves simultaneously [18]. Thus, we employed modal class assignment at each time and calculated transition probabilities (i.e., probability of class assignment at follow-up conditional on class assignment at baseline) without imposing the restriction that class interpretations are identical across waves.

Table 5 shows the association between health and well-being (assigned) class membership at baseline and at follow-up. For example, among individuals assigned to the Healthy, Low-Risk class at baseline, only 56.9% were expected to be in that class at follow-up whereas 38.6% were expected to move into the Depressed class. Among students in the Depressed class at baseline, three-quarters were expected to be in that class at follow-up and 22.5% expected to move to the Healthy, Low-Risk class. Students in the Poor Mental Health, Sex, Food Insecure class at baseline were most likely (56.9%) to be in the Depressed class at follow-up; this suggests that mental health challenges were likely to persist during the pandemic, but these students became less sexually active and had greater food insecurity. Of those in the Partiers (Alcohol, Sex) class at baseline, during the pandemic approximately one-third were in the Depressed class and nearly one-third were in the Healthy, Low Risk class. Very few (8.5%) maintained membership in the Partiers (Alcohol, Sex) class at follow-up. Individuals in the Partiers (Polydrug, Sex) class at baseline were fairly equally distributed across all classes at follow-up (all probabilities less than 25%). Finally, students in the Highest Risk class at baseline had the highest chance of being in the Poor Mental Health, Heavy Alcohol Use class at follow-up (39.1%).

**Table 5:** Approximate cell counts and probabilities of membership in health and well-being class at follow-up conditional on baseline class membership.

	Latent class membership at follow-up					
Latent class membership at baseline (percentage expected in class)	Depressed	Healthy, Low-Risk	Poor Mental Health, Heavy Alcohol Use	Depressed, Cannabis Use, Sex	Partiers (Alcohol, Sex)	Poor Mental Health, Food Insecure, Low Physical Activity
Healthy, Low-Risk (35.9%)	164 (38.6%)	**242** **(56.9%)**	5 (1.2%)	5 (1.2%)	8 (1.9%)	1 (0.2%)
Depressed (24.8%)	**159** **(74.7%)**	48 (22.5%)	1 (0.5%)	2 (0.9%)	1 (0.5%)	2 (0.9%)
Poor Mental Health, Sex, Food Insecure (15.1%)	**83** **(56.9%)**	17 (11.6%)	13 (8.9%)	14 (9.6%)	1 (0.7%)	18 (12.3%)
Partiers (Alcohol, Sex) (10.7%)	**36** **(38.3%)**	28 (29.8%)	13 (13.8%)	8 (8.5%)	8 (8.5%)	1 (1.1%)
Partiers (Polydrug, Sex) (8.2%)	15 (19.0%)	15 (19.0%)	13 (16.5%)	**19** **(24.1%)**	15 (19.0%)	2 (2.5%)
Highest Risk (5.4%)	14 (30.4%)	2 (4.4%)	**18** **(39.1%)**	9 (19.6%)	2 (4.4%)	1 (2.2%)

*Notes*. (1) Percentages across rows sum to 100%. (2) For each baseline class, most likely latent class membership at follow-up denoted in bold font. (3) For this table, modal class assignment was used (i.e., at each wave, individuals are assigned to class they most likely belong to given responses to set of 10 health and well-being indicators). Class membership uncertainty was not taken into account, thus cell counts and transition probabilities are approximate.

### Covariates of well-being profiles

Omnibus tests for the association between each covariate and baseline latent class membership revealed that well-being class membership *prior to the COVID-19 pandemic* indicated significant associations with sex, sexual or gender minority status, racial/ethnic group, and feelings of not belonging at the university (see Table 6.; The 3-step BCH approach [34] is the preferred method to examine associations between covariates and class membership, however estimation problems with these specific models prohibited its use. We therefore relied on modal class assignment with subsequent chi-square and ANOVA tests. This approach yields conservative tests of associations between covariates and class membership.). Students in the Poor Mental Health, Sex, Food Insecure class and the Highest Risk class reported the highest levels of feelings of not belonging at the University and comprised the lowest percentages of males and the highest percentages of sexual or gender minority students. Students in either Partiers class were more likely than students in other classes to be non-Hispanic white. Students in the Healthy, Low-Risk class and the two Partiers classes reported the lowest levels of feelings of not belonging.

**Table 6:** Covariates of health and well-being latent class membership prior to the pandemic: Class-specific proportion (mean) of individuals with each characteristic at baseline.

Baseline covariate	Test of overall association^a^	Healthy, Low-Risk	Depressed	Poor Mental Health, Sex, Food Insecure	Partiers (Alcohol, Sex)	Partiers (Poly-Drug, Sex)	Highest Risk
Male	****	0.439	0.366	0.145	0.479	0.532	0.217
Sexual or gender minority	****	0.071	0.189	0.227	0.106	0.051	0.283
Upper-class standing	*ns*	0.226	0.249	0.315	0.266	0.203	0.217
First-generation student	*ns*	0.272	0.360	0.312	0.255	0.256	0.391
Race/ethnicity	*						
Hispanic/Latinx		0.075	0.076	0.121	0.044	0.053	0.070
NH Black		0.052	0.106	0.086	0.033	0.026	0.047
NH Asian		0.122	0.141	0.114	0.065	0.040	0.093
NH white		0.751	0.677	0.679	0.859	0.882	0.791
Feelings of not belonging prior to pandemic: *M* (*95% confidence interval*)	****	0.87 (0.78, 0.96)	1.38 (1.25, 1.51)	1.67 (1.51, 1.82)	1.27 (1.08, 1.46)	0.97 (0.77, 1.18)	1.78 (1.51, 2.05)

*Notes*. NH = Non-Hispanic/Latinx. Significance levels denoted as

* *p* < .05,

** *p* < .01,

*** *p* < .001,

**** *p* < .0001, *ns* = not statistically significant at *p* < .05.

^a^ Tests of association based on chi-square test statistic for categorical covariates, overall ANOVA *F* test statistic (4 *df*) for continuous covariate.

Omnibus tests for the association between each covariate and baseline latent class membership revealed that well-being class membership *during the COVID-19 pandemic* indicated significant associations with numerous sociodemographic characteristics at baseline, including sex, sexual or gender minority status, upper-class standing, and racial/ethnic group (see Table 7). Additional characteristics assessed at follow-up were significantly associated with well-being profiles, including mechanisms for coping with the pandemic (specifically, healthy behaviors, smoking/vaping more, drinking alcohol, using cannabis, and eating high-fat or sugary foods), practicing consistent social distancing, not living with parents, and feelings of not belonging at the university.

**Table 7:** Covariates of health and well-being latent class membership during the pandemic: Class-specific proportion (mean) of individuals with each characteristic at follow-up.

Covariate	Test of Overall Assoc-iation^a^	Depressed	Healthy, Low-Risk	Poor Mental Health, Heavy Alcohol Use	Depressed, Cannabis Use, Sex	Partiers (Alcohol, Sex)	Poor Mental Health, Food Insecure, Low PA
***Baseline Covariates***							
Male	****	0.319	0.464	0.413	0.281	0.600	0.250
Sexual or gender minority	****	0.179	0.049	0.159	0.143	0.057	0.348
Upper-class standing	****	0.198	0.247	0.444	0.263	0.343	0.440
First-generation student	*ns*	0.306	0.269	0.318	0.316	0.382	0.381
Race/ethnicity	****						
Hispanic/Latinx		0.097	0.048	0.033	0.091	0.029	0.250
NH Black		0.077	0.066	0.000	0.036	0.000	0.125
NH Asian		0.140	0.117	0.033	0.018	0.000	0.083
NH white		0.686	0.769	0.934	0.855	0.971	0.542
***Covariates at Follow-Up***							
Coping during Pandemic							
Healthy behaviors	****	0.612	0.739	0.698	0.632	0.829	0.320
Connect socially	*ns*	0.724	0.759	0.810	0.737	0.829	0.840
Smoke/vape more	****	0.006	0.000	0.191	0.298	0.114	0.200
Drinking alcohol	****	0.123	0.065	0.794	0.526	0.571	0.080
Using cannabis	****	0.038	0.017	0.222	0.509	0.171	0.280
High-fat/sugary food	****	0.461	0.276	0.587	0.404	0.343	0.480
Consistent social distance	**	0.443	0.430	0.210	0.351	0.286	0.458
Not living with parents	****	0.096	0.094	0.333	0.140	0.086	0.560
Feelings of not belonging during pandemic: *M* (*SD*)	****	1.27 (1.18, 1.35)	0.77 (0.67, 0.86)	1.30 (1.07, 1.53)	1.44 (1.20, 1.68)	0.51 (0.21, 0.82)	1.48 (1.12, 1.84)

*Notes*. NH = Non-Hispanic/Latinx. Significance levels denoted as

* *p* < .05,

** *p* < .01,

*** *p* < .001,

**** *p* < .0001, *ns* = not statistically significant at *p* < .05.

^a^ Tests of association based on chi-square test statistic (4 *df*) for categorical covariates, overall ANOVA *F* test statistic (4 *df*) for continuous covariate.

As shown in Table 7, students in the Poor Mental Health, Food Insecure, Low PA class comprised the lowest percentage of males and the highest percentage of sexual or gender minority students, Hispanic students, and non-Hispanic Black students. Students in both latent classes characterized by poor mental health were most likely to comprise upper-class students. Students in the Partiers (Alcohol, Sex) class had the highest percentage of non-Hispanic white students. Not surprisingly, students in the Depressed with Cannabis Use and Sex class were much more likely to report coping by using cannabis compared to students in any other class. Students in the Partiers class were most likely to endorse coping via healthy behaviors; these students had the highest proportion of males and the lowest feelings of not belonging. Students in the Poor Mental Health, Heavy Alcohol Use class were very likely to report coping via drinking alcohol and also eating high-fat or sugary foods, and were the least likely to endorse consistent social distancing practices. More than half of students in the Poor Mental Health, Food Insecure, Low PA class reported not living with their parents. These students were the most likely to report practicing consistent social distancing, the least likely to report coping with the pandemic via healthy behaviors and (along with students in the Depressed with Cannabis Use and Sex class) the highest level of feelings of not belonging at the university.

The Depressed subgroup comprised the highest proportion of students early in their education (i.e., they had the lowest proportion of students with upper-class standing). Finally, the Healthy, Low-Risk subgroup had the lowest proportion of sexual and gender minority students and were least likely to endorse smoking, drinking alcohol, using cannabis, or eating high-fat or sugary foods to cope with the pandemic. Students in the Healthy, Low-Risk class and the Partiers class had the highest probabilities of using healthy behaviors to cope.

## Discussion

Rather than consider a single indicator of college student well-being, this study considered ten intersecting indicators of health and well-being just before and during the COVID-19 pandemic. Somewhat consistent with previous research [15,35], we found significant increases in some reports of depressive symptoms, reductions in substance use, reductions in physical and sexual activity, and reductions in food insecurity (Table 1). In concert with prior work examining changes in perceived norms and motivations for use [36]–particularly increases in using substances to cope [37]–it is clear that psychosocial risk factors for and prevalence of health behaviors have evolved across the pandemic. A critical contribution of the current study was the use of LCA to consider a broad set of indicators of health and well-being simultaneously, at each timepoint, and examine change over time. Just before the pandemic, two very large subgroups emerged: one group with a very Healthy, Low-Risk profile comprising 35.9% of students, and another group (Depressed) with a similar low-risk profile with the exception of elevated depression symptoms that comprised 24.8% of students. This calls attention to the fact that, even among the large majority of students who had very few indicators of poor well-being, the rate of students experiencing depression symptoms after the onset of the pandemic was incredibly high. The remaining 39% of students comprised four relatively small but distinct groups of students defined by their well-being profiles. Two of these groups were characterized by risky alcohol use, yet the Poor Mental Health, Sex, Food Insecure class comprised students with considerable mental health problems whereas Partiers (Alcohol, Sex) was largely absent of mental health problems. Yet another group—Partiers (Polydrug, Sex)—engaged in polydrug use and endorsed this behavior as a mechanism for coping with the ongoing pandemic. Most concerning, perhaps, was the small group of students comprising the Highest Risk group. These students experienced very high rates of mental health problems, substance use, and likely had food insecurity and minimal physical activity. These intersecting health challenges place the group at high risk for poor academic, social, and health outcomes. In the same vein, our examination of covariates associated with health and well-being profiles during the pandemic revealed that many students in this group had important characteristics that place them at risk, such as being female, a racial/ethnic minority student, a sexual or gender minority student, and having low feelings of belonging at the university, consistent with prior work investigating the effects of COVID-19 on health disparity populations [9,11,17,19–22,38]. Nearly three-quarters of these students reported that they did not live with their parents during the pandemic, likely contributing to their heightened levels of food insecurity during an unusually challenging time in history.

Several interesting findings emerged when examining latent classes during the pandemic. Three of the classes detected pre-pandemic–Healthy, Low-Risk; Depressed; and Partiers (Alcohol, Sex)—were similar at follow-up, however three new classes emerged: the Poor Mental Health, Heavy Alcohol Use class (characterized by high-intensity alcohol use); the Depressed, Cannabis Use, Sex class; and the Poor Mental Health, Food Insecurity, Low Physical Activity class. Key to these findings are the transitions we observed between baseline and follow-up. Importantly, only 57% of participants who were in the Healthy, Low-Risk class at baseline were expected to remain in this class during the pandemic. Despite the short time window between baseline and follow-up (approximately 5 months), this finding highlights the sudden and heterogeneous impact of the COVID-19 pandemic on college students.

A national report on basic needs insecurity based on nearly 200,000 students from across the nation suggests that 29% of four-year college students faced food insecurity during the Fall 2020 semester [39]. In our population, 33.0% of students reported food insecurity in November 2019, just before the pandemic, compared to 20.5% in May 2020 when most students had moved home. The astounding rate of food insecurity (0.974; see Table 4) among students in the Poor Mental Health, Food Insecure, Low PA group indicate that structural/institutional interventions for such students are greatly needed.

Several limitations should be noted in the current study. First, substance use and other health behaviors were based on participants’ self-reports, which may be impacted by social desirability or recall bias. Second, our sample is primarily non-Hispanic white, which limits generalizability to other racial-ethnic groups, particularly Black and Latinx college students. Third, results were based on college student behavior at a single university in the northeastern region of the US; findings may not generalize to other regions of the country and/or other universities.

The multi-dimensional profiles of college student well-being identified in this study convey how critical it is to consider students in a comprehensive manner when considering what services and interventions would be most useful to their success. Certainly, these results suggest the importance of elevated depressive symptoms among college students–indeed, four of the six profiles are marked by this indicator. The only groups of college students that did not endorse elevated depression symptoms during the pandemic were those in the Healthy, Low-Risk group and the Partiers group. Yet the services indicated by students suffering from depression symptoms are varied. For many students, this was the primary challenge they faced during the pandemic; for others, elevated depression symptoms intersected with different combinations of heightened anxiety symptoms, risky alcohol use, cannabis use, lack of physical activity, and/or food insecurity. Taking the broader student experiences into consideration can inform more efficient, tailored supports for college students that meet them where they are, with the services that they need. These findings are particularly salient in light of emerging longitudinal evidence findings that the effects of COVID-19 have direct and enduring impacts on mental health and well-being (e.g., [36]).

## Conclusions

Prevalence and transitions in subgroup membership before and during the start of the pandemic indicate that mental health challenges largely persisted, despite other health risks lessening. Our findings convey the enormous role the pandemic has played on the mental health of college students and reveal key predictors of mental health during this time (e.g., belongingness, racial/ethnic group, sexual/gender minority status). By examining the intersection of a wide range of health and well-being indicators—including of mental health, substance use, physical inactivity, and food insecurity—smaller groups of students were identified with an indicated need for more comprehensive services. Efforts to consider the broad health effects of the recent pandemic, and stress more broadly, among college students offer opportunities to tailor university services to improve student outcomes and reduce health disparities.

## Supporting information

S1 Appendix Complete survey instrument used at Wave 1. (DOCX)

S2 Appendix Complete survey instrument used at Wave 2. (DOCX)
