# Supplementary material for: Change in college student health and well-being profiles as a function of the COVID-19 pandemic
Source: PLoS One. 2022 May 2;17(5):e0267724. doi: 10.1371/journal.pone.0267724 (PMC9060353; doi:10.1371/journal.pone.0267724)
Supplement: S1 Appendix — (DOCX) [file pone.0267724.s001.docx]

**S1 Appendix. Survey instrument used at Wave 1**

**Wave 2 Screening Questionnaire**

1. What Penn State Campus do you attend?

- University Park
- Abington
- Greater Allegheny
- Beaver
- Berks
- Altoona
- Behrend
- Harrisburg
- Brandywine

2. What year are you in school?

- First year or Second year
- Third year or later
- How old are you? Under 18
- 18-24
- 25 or older

**Wave 2 Questionnaire**

1. What is your enrollment status at Penn State this semester?

- Full-time (taking 12 or more credits)
- Part-time (taking less than 12 credits)
- Prefer not to answer

1. What is your sex that was assigned at birth? (For example, on your birth certificate)

- Male
- Female
- Intersex
- Prefer not to say

1. What is your current gender?

- Male/man
- Female/woman
- Genderqueer/gender nonconforming
- Trans male/trans man
- Trans female/trans woman
- Different identity: [write in]
- Prefer not to say

1. Which option best describes your sexual identity/orientation?
   - Heterosexual/straight
   - Bisexual
   - Gay
   - Lesbian
   - Queer
   - None of these options apply (please describe) [write-in]
   - Prefer not to say
2. Have you been sexually active in the past month?
   - Yes (go on to the next question)
   - No (skip the next question)
   - Prefer not to answer
3. How many sexual partners have you had?
   - One
   - Two
   - Three or more
   - Prefer not to answer
4. How old are you?

8. Is your home residence (when not in school)

- In Pennsylvania
- In the US, but a different state other than Pennsylvania
- Outside of the US (international student)
- Prefer not to answer

9. Your Height (Please enter: feet.inches (e.g., 5.2 for 5’2” or 5.11 for 5’11”; If you prefer not to answer, please type in 9.99) : ______ feet ______ inches

10. Your Current Weight (in lbs; If you prefer not to answer, please type in 999) : _______ lbs.

Weight in pounds is equivalent to the value of their weight – example 300 pounds it is“300”

11. What is your race or ethnicity? Mark all that apply.

- White
- Hispanic, Latino/a, or Spanish
- Black or African American
- Asian
- Native American or Alaska Native
- Middle Eastern or North African
- Native Hawaiian or Other Pacific Islander
- Some other race or ethnicity [write in please specify]
- Decline to answer

12. Please indicate whether you’re currently involved in the following groups:

| **Group Name** | **Yes** | **Maybe in the Future** | **No** | **Prefer not to answer** |
| --- | --- | --- | --- | --- |
| THON Organizations |  |  |  |  |
| Schreyer Honors College |  |  |  |  |
| Academic Group (e.g., research lab, departmental committee) |  |  |  |  |
| Government/political/activism group |  |  |  |  |
| Cultural/ethnic/religious group |  |  |  |  |
| Creative group (e.g., performing arts, journalism) |  |  |  |  |
| Greek- academic |  |  |  |  |
| Greek- social |  |  |  |  |
| Sport (club, intramural, intercollegiate/varsity) |  |  |  |  |

13. What is your current college standing?

- First year
- Second year
- Third year
- Fourth year
- Other

14. Were you a part of the LEAP program at Penn State (Start at PSU the summer before first fall at Penn State)?

- Yes
- No
- Prefer not to answer

15. What was your High School grade point average (GPA)? [Please enter a number with one decimal place. (e.g., 3.5) (If you prefer not to answer, please type in 9.9)

_____________

16. Are you employed during the academic year?

- Yes, I work full time
- Yes, I work part time
- No I do not work
- Prefer not to answer

17. Where do you currently live during the academic year?

- College dorm/residence hall
- House/apartment/room (not college-affiliated)
- Parent’s or relative’s home
- Fraternity/sorority house
- Other
- Prefer not to answer

18. What was the highest level of education completed by your Mother?

- Do not know mother’s education level
- Did not complete high school
- High school diploma or equivalent
- Vocational/technical training
- Associate’s degree
- Master’s degree or equivalent
- Doctoral degree- (PhD, MD)
- Prefer not to answer

19. What is the highest level of education completed by your Father?

- Do not know mother’s education level
- Did not complete high school
- High school diploma or equivalent
- Vocational/technical training
- Associate’s degree
- Master’s degree or equivalent
- Doctoral degree— (PhD, MD)
- Prefer not to answer

20. Were you born in the United States?

- Yes
- No
- Don’t know
- Prefer not to answer

21. Was your mother born in the United States?

- Yes
- No
- Don’t know
- Prefer not to answer

24. Was your father born in the United States?

- Yes
- No
- Don’t know
- Prefer not to answer

25. In the past month, how many times have you experienced bias or discrimination?

- Always
- Frequently
- Sometimes
- Hardly ever
- Never
- Prefer not to answer

26. I feel like I belong at Penn State

- Strongly Agree
- Agree
- Slightly Agree
- Neither agree nor disagree
- Slightly disagree
- Disagree
- Strongly disagree
- Prefer not to answer

27. When you think about Penn State, how often, if ever, do you wonder: “Maybe I don’t belong here?”

- Always
- Frequently
- Sometimes
- Hardly ever
- Never
- Prefer not to answer

28. Within the past month, I worried whether my food would run out before I got money to buy more.

- Often true,
- Sometimes true
- Never true.
- Prefer not to answer

29.Within the past month, the food I bought just did not last and I did not have money to get more.

- Often true,
- Sometimes true
- Never true.
- Prefer not to answer

30. In the past 30 days, have you had any appointments with on campus health services (for physical or mental health)?

- Yes (go to 30a and 30b)
- No (go to 31)
- Prefer not to answer

30a. How many appointments were primarily for physical health? ___ (enter number)

30b. How many appointments were primarily for mental health/counseling? _____ (enter number)

31. In the past 30 days have you seen a health care provider OFF campus (for physical or mental health)?

- Yes
- No
- Prefer not to answer

32. For the purpose of this questionnaire, being physically active means doing activities such as strength training (e.g., weightlifting, swimming, running), playing sports, outdoor activities (e.g., hiking, skiing) for at least 30 minutes, 3 times a week

Think about the past month, have you met these guidelines:

- Every week
- Some weeks
- No weeks
- Prefer not to answer

33**.** During the last week….

(Scale- 0-Rarely or none of the time, to 3-most or all of the (time with a prefer not to answer for each)

I was bothered by things that usually don’t bother me

I had trouble keeping my mind on what I was doing

I felt depressed

I felt that everything I did was an effort

I felt hopeful about the future

I felt fearful

My sleep was restless

I was happy

I felt lonely

I could not get “going”

34. Anxiety subscale from CCAPS-34.

Scale: 0- not at all like me to 4- extremely like me (with a prefer not to answer for each)

35. Have you ever tried alcoholic beverage- more than just a few sips?

- Yes, in the last 30 days [Go to question 36 for males, 38 females]
- Yes, but not in the last 30 days [Go to question 36 for males, 38 females]
- No [Go to question 40]
- Prefer not to answer [Go to question 40]

One Drink equals:
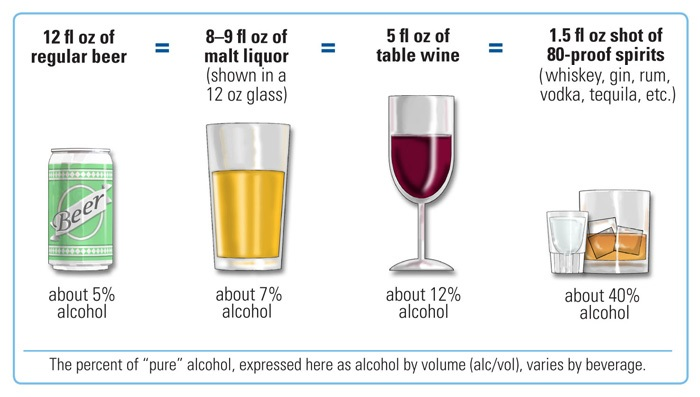


- 12oz of beer (a typical plastic party cup holds 16oz)
- 5oz of wine
- 1.5oz shot of 80 proof liquor or 1 oz shot of 100 proof liquor

36. [Male] Have you ever consumed 5 or more alcoholic drinks in one sitting?

- No [Go to question 40]
- Yes, but not within the last 30 days [Go to question 40]
- Yes, within the past 30 days [Go to question 37]
- Prefer not to answer [Go to question 40]

37. [Male] Have you ever consumed 10 or more alcoholic drinks in one sitting?

- No [Go to question 40]
- Yes, but not within the last 30 days [Go to question 40]
- Yes, within the past 30 days [Go to question 40]
- Prefer not to answer [Go to question 40]

38. [Female] Have you ever consumed 4 or more alcoholic drinks in one sitting?

- No [Go to question 40]
- Yes, but not within the last 30 days [Go to question 40]
- Yes, within the past 30 days [Go to question 39]
- [prefer not to answer [Go to question 40]

39. [Female] Have you ever consumed 8 or more alcoholic drinks in one sitting?

- No
- Yes, but not within the last 30 days
- Yes, within the past 30 days
- Prefer not to answer

40. Have you ever used any kind of nicotine or tobacco product?

- Yes [Go to question 41]
- No [Go to question 42]
- Prefer not to answer [Go to question 42]

41. Which nicotine or tobacco products have you used (check all that apply)?

|  | Tried, but not in past 30 days | Used in the last 30 days | Never |
| --- | --- | --- | --- |
| Cigarette |  |  |  |
| Vaping /E-cigarettes |  |  |  |
| Cigarillo/cigar |  |  |  |
| Hookah |  |  |  |
| Smokeless tobacco product (e.g., chew, snus |  |  |  |
| Nicotine replacement product (e.g., patch, gum) |  |  |  |

42. Have you ever used marijuana or hashish?

- Yes, within the last 30 days
- Yes, but not within the last 30 days
- No
- Prefer not to answer

43. Have you ever used any of the following: (check all the apply)

- Prescription stimulants for NON-medical purposes (e.g., Ritalin, Adderall)
- Prescription painkillers for NON-medical purposes (e.g., oxycontin, Vicodin)
- Other prescription drugs for NON-medical purposes (e.g., Xanax)
- Psychedelics (e.g., LSD, Ecstasy, Molly, mushrooms)
- Any other illicit drug (e.g., cocaine, meth)
- I have NOT used any of the above substances
- Prefer not to answer

44. What type of smartphone do you own?

- Apple iPhone (any model)
- Samsung Galaxy (any model)
- Google Pixel (any model)
- OnePlus Pro (any model)
- Huawei Pro (any model)
- Other Smartphone
- I do not own a Smartphone
- Prefer not to answer

45. Do you have any other Penn State specific experience that you’d like to tell us about?
