# Supplementary material for: Change in college student health and well-being profiles as a function of the COVID-19 pandemic
Source: PLoS One. 2022 May 2;17(5):e0267724. doi: 10.1371/journal.pone.0267724 (PMC9060353; doi:10.1371/journal.pone.0267724)
Supplement: S2 Appendix — (DOCX) [file pone.0267724.s002.docx]

**S2 Appendix. Survey instrument used at Wave 2**

**Wave 2 Screening Questionnaire**

What was your enrollment status at Penn State during the Spring 2020 semester?

- Full-time (taking 12 or more credits)
- Part-time (taking less than 12 credits)
- Not enrolled

**Wave 2 Questionnaire**

1. What is your biological sex?
   - Male
   - Female
   - Prefer not to answer
2. How old are you?
3. What is your employment status *today*?

- I lost my job or am furloughed due to the COVID-19 pandemic
- I do not work for pay
- I have a full-time job but not currently working due to stay at home guidelines
- I have one or more part-time jobs but not currently working due to stay at home guidelines
- I have a full-time job and am currently working
- I have one part-time job and am currently working
- I have more than one part-time job and am currently working
- Other (specify)_______
- Prefer not to answer

1. Have you been back to campus for any reason since the governor began issuing stay at home orders on March 23^rd^ 2020?

- Yes
- No
- Prefer not to answer

1. The chances of my getting COVID-19 are high

- - - Strongly disagree
    - Disagree
    - Not sure
    - Agree
    - Strongly Agree
    - Prefer not to answer

1. Have you been tested for COVID-19?

- No [Go to question 10]
- Yes
- Prefer not to answer [Go to question 10]

1. What was the test result and outcome?

- Tested negative for COVID-19 [Go to question 9]
- Tested positive and have since recovered
- Tested positive but have not fully recovered
- Prefer not to answer  [Go to question 10]

*For those who tested positive:*

1. How negatively was your health affected by the COVID-19 virus?

- Not at all affected
- A little affected
- Mildly affected
- Moderately affected
- Very negatively affected
- Prefer not to answer

[Go to Question 11]

*For those who tested negative:*

1. If you were to be infected with the COVID-19 virus, how negatively do you think your health would be affected?

- Not at all affected
- A little affected
- Mildly affected
- Moderately affected
- Very negatively affected
- Prefer not to answer

*For those not tested and those that tested negative:*

1. Since January 2020, have you had symptoms of COVID-19 (dry cough, fever, shortness of breath)?

- I have not had any symptoms
- I have had mild symptoms
- I have had somewhat severe symptoms
- I have had moderately severe symptoms
- I have had severe symptoms
- Prefer not to answer

1. Have you *ever* been in close proximity (6 feet or less) to someone showing key symptoms of COVID-19 (fever, cough, shortness of breath) or who tested positive for COVID-19?

- No [Go to question 13]
- Yes
- Prefer not to answer

*If YES:*

1. During the past two weeks, have you been in close proximity to someone showing key symptoms of COVID-19 (fever, cough, shortness of breath) or who tested positive for COVID-19?

- No
- Yes
- Prefer not to answer

1. Individuals vary in their ability and interest in practicing “social distancing,” that is, maintaining at least 6 feet between themselves and others (not including those they live with).  Using the scale below, please indicate how often you have practiced social distancing *during the past two weeks*.

- - Not at all
  - 2
  - 3
  - Neutral
  - 5
  - 6
  - All of the time
  - Prefer not to answer

1. *During the past two weeks*, how consistently have you worn a scarf or facial mask when in public (that is, when there is a chance of being within 6 ft of individuals who do not live with you – such as when grocery shopping)?
   - Not at all
   - 2
   - 3
   - Neutral
   - 5
   - 6
   - Always
   - Prefer not to answer
2. *During the past two weeks*, how consistently have you practiced handwashing according to current guidelines (regularly wash hands with warm water and soap for 20 seconds)?

- - Not at all
  - 2
  - 3
  - Neutral
  - 5
  - 6
  - All of the time
  - Prefer not to answer

1. How informed are you about the current Covid-19 situation?
   - Not informed at all
   - 2
   - 3
   - Neutral
   - 5
   - 6
   - Very Informed
   - Prefer not to answer
2. How often do you get information on the coronavirus pandemic from the following sources:

Radio buttons 1-7 (Never- Frequently during the day, with prefer not to answer as 8)

- Internet such as websites
- Social media, such as Facebook and Twitter
- Radio
- Television
- Printed newspapers
- Printed magazines
- Friends or family members
- Doctor or other healthcare providers
- Other, please specify_________
- Prefer not to answer

1. To cope with social distancing and isolation, are you doing any of the following? (check all that apply)

- Taking breaks from watching, reading, or listening to news stories
- Taking deep breaths, stretching, or meditating
- Engaging in healthy behaviors (e.g., eating healthy meals, exercising regularly, getting plenty of sleep)
- Making time to relax
- Making efforts to socially connect with friends (e.g., Zoom, FaceTime, Netflix Party, etc.)
- Contacting a counselor/mental healthcare provider
- Smoking more cigarettes or vaping more
- Drinking alcohol
- Using cannabis/marijuana
- Using other drugs
- Eating high fat or sugary foods
- None of the above
- Prefer not to answer

1. Which of the following best describes your life’s *disruption* due to coronavirus?

- My life has been significantly disrupted
- My life has been moderately disrupted
- My life has not been disrupted
- Prefer not to answer

1. What do you think of the actions taken by your government or local health authority to prevent and/or reduce the spread of coronavirus (which causes the disease COVID-19)?

- Too strict
- About right
- Too lenient
- I don’t know/I prefer not to answer

1. If a vaccine for COVID-19 were available today, what is the likelihood that you would get vaccinated?

- Extremely likely
- Somewhat likely
- Unsure
- Unlikely
- Very unlikely
- Prefer not to answer

1. In the past month, how many times have you experienced bias or discrimination?

- Always
- Frequently
- Sometimes
- Hardly ever
- Never
- Prefer not to answer

1. I feel like I belong at Penn State
   - Strongly Agree
   - Agree
   - Slightly Agree
   - Neither agree nor disagree
   - Slightly disagree
   - Disagree
   - Strongly disagree
   - Prefer not to answer
2. When you think about Penn State, how often, if ever, do you wonder: “Maybe I don’t belong here”?

- Always
- Frequently
- Sometimes
- Hardly ever
- Never
- Prefer not to answer

1. Within the past month, I worried whether my food would run out before I got money to buy more.

- Often true,
- Sometimes true
- Never true.
- Prefer not to answer

1. Within the past month, the food I bought just did not last and I did not have money to get more.

- Often true,
- Sometimes true
- Never true.
- Prefer not to answer

1. In the past 30 days, did you NOT pay or underpay your rent?

- Yes
- No
- Prefer not to answer

1. In the past 30 days, did you NOT pay the full amount of a gas, oil, or electricity bill?

- Yes
- No
- Prefer not to answer

1. In the past 30 days, have you moved two times or more?

- Yes
- No
- Prefer not to answer

1. In the past 30 days, did you live with others beyond the expected capacity of the

house or apartment?

- - Yes
  - No
  - Prefer not to answer

1. Since starting college, have you ever been homeless (homeless meaning: lacking a fixed, regular and adequate nighttime residence)?
   - Yes
   - No [Go to question 33]
   - Prefer not to answer [Go to question 33]

*If they answered “yes” to ever being homeless:*

1. Please specify the timing of your homelessness since starting college:
   - Both before and after the COVID pandemic
   - Only before the pandemic
   - Only after the pandemic
   - Prefer not to answer
2. In the past 30 days, have you slept in any of the following places? Please mark

all that apply.

- - Campus or university housing
  - Sorority/fraternity house
  - Alone in a rented or owned house, mobile home, or apartment
  - With roommates or friends in a rented or owned house, mobile home, or apartment
  - With family (parent, guardian, relative) in a rented or owned house, mobile home, or apartment with my family
  - Temporarily staying with a relative, friend, or couch surfing until I find other housing
  - At a shelter
  - In a camper
  - Temporarily at a hotel or motel without a permanent home to return to (not on vacation or business travel)
  - In transitional housing or independent living program
  - At a group home (such as halfway house or residential program for mental health or substance abuse)
  - At a treatment center (such as detox, hospital, etc.)
  - Outdoor location (such as street, sidewalk, or alley, bus or train stop, campground or woods, park, beach, or riverbed, under bridge or overpass)
  - In a closed area/space with a roof not meant for human habitation (such as abandoned building, car, truck, van, tent, or unconverted garage, attic, or basement)
  - Prefer not to answer

1. In the past two weeks have you experienced the following as a result of COVID-19?

- Not enough money to pay rent
- Not enough money to pay for gas
- Not enough money to pay for food
- Did not have a regular place to sleep or stay
- I have not experienced any of the above
- Prefer not to answer

1. In the past two weeks has your family experienced the following as a result of COVID-19?

- Not enough money to pay rent
- Not enough money to pay for gas
- Not enough money to pay for food
- Did not have a regular place to sleep or stay
- My family has not experienced any of the above
- Prefer not to answer

1. Are you aware that Penn State’s Student Care and Advocacy provides a student emergency fund for students experiencing financial difficulties?
   - Yes
   - Yes, I have used the fund since the University went to remote learning
   - No 🡪 if NO, display link to info about emergency fund [<https://studentaffairs.psu.edu/studentcare>]
   - Prefer not to answer
2. Who are you living with today? Check all that apply.

- Spouse/significant other
- Children under your care
- Parents, caregivers, stepparents, etc.
- Extended family (such as aunts, uncles, grandparents)
- Brothers or sisters
- Friends
- Currently live alone [Go to question 39]
- Currently homeless [Go to question 39]
- Other [specify]
- Prefer not to answer

*If not currently living alone or homeless:*

1. Thinking of who you live with currently: Over the past two weeks, how true are the following statements?

Scale of 1-7 (Never true to Always true, with 8 being Prefer not to answer)

- Household members really help and support one another
- There is a feeling of togetherness in our household
- We really get along well with each other
- We fight a lot in our household
- Household members hardly ever lose their tempers
- Household members often criticize each other

1. Who do you identify as caregiving adults in your life? Mark all that apply.

- Mother
- Second mother
- Father
- Second father
- Stepmother
- Stepfather
- Aunt
- Uncle
- Grandmother
- Grandfather
- Mother's partner
- Father's partner
- Foster mother
- Foster father
- Other caregiver
- No caregiving adults [Go to question 41]
- Prefer not to answer

1. Thinking generally about any caregiver you have, please indicate the extent to which the following statements are true over the past two weeks. Even if you don’t live with them, please respond to these questions based on your experiences with them, in terms of your opinion of the relationship and interactions you have by phone, text, or messaging.

Scale of 1-7 (Never true to Always true, with 8 being Prefer not to answer)

- My caregiver respects my feelings.
- I tell my caregiver about my problems and troubles.
- My caregiver encourages me to talk about my difficulties.
- I feel close to caregiver.
- The talks we have are frustrating.
- We argued or disagreed with each other.
- We haven’t gotten along well.

1. For the purpose of this questionnaire, being physically active means doing activities such as strength training (e.g., weightlifting, swimming, running), playing sports, outdoor activities (e.g., hiking, skiing) for at least 30 minutes, 3 times a week.

Think about the past month, have you met these guidelines:

- Every week
- Some weeks
- No weeks
- Prefer not to answer

1. Have you been sexually active in the past month?
   - Yes
   - No [Go to question 44]
   - Prefer not to answer
2. How many sexual partners have you had in the last month?
   - One
   - Two
   - Three or more
   - Prefer not to answer
3. During the last week….

(Scale- 0-Rarely or none of the time to 3- Most or all of the time) All have a prefer not to answer option

- I was bothered by things that usually don’t bother me
- I had trouble keeping my mind on what I was doing
- I felt depressed
- I felt that everything I did was an effort
- I felt hopeful about the future
- I felt fearful
- My sleep was restless
- I was happy
- I felt lonely
- I could not get “going”

.

1. Anxiety subscale from CCAPS-34

(Scale: 0- not at all like me to 4- extremely like me) All have a prefer not to answer option

1. Have you ever tried an alcoholic beverage, more than just a few sips?

- Yes, in the last 30 days [Go to question 47 for males, 49 females]
- Yes, but not in the last 30 days [Go to question 47 for males, 49 females]
- No [Go to question 51]
- Prefer not to answer [Go to question 51]

One Drink equals:
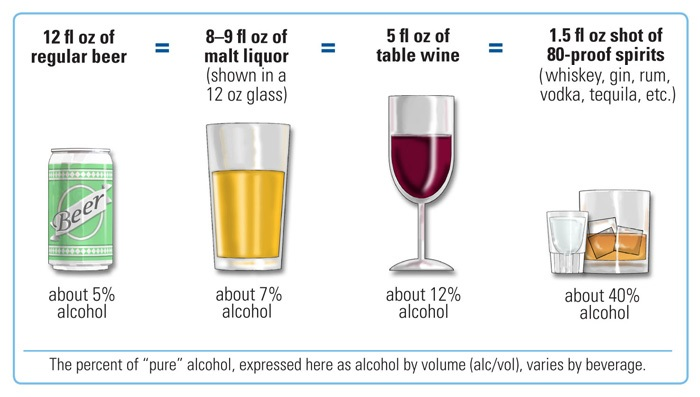


1. 12oz of beer (a typical plastic party cup holds 16oz)
2. 5oz of wine
3. 1.5oz shot of 80 proof liquor or 1 oz shot of 100 proof liquor
4. [Male] Have you ever consumed 5 or more alcoholic drinks in one sitting?

- No [Go to question 51]
- Yes, but not within the last 30 days [Go to question 48]
- Yes, within the past 30 days [Go to question 48]
- Prefer not to answer [Go to question 51]

1. [Male] Have you ever consumed 10 or more alcoholic drinks in one sitting?

- No [Go to question 51]
- Yes, but not within the last 30 days [Go to question 51]
- Yes, within the past 30 days [Go to question 51]
- Prefer not to answer [Go to question 51]

1. [Female] Have you ever consumed 4 or more alcoholic drinks in one sitting?

- No [Go to question 51]
- Yes, but not within the last 30 days
- Yes, within the past 30 days
- [prefer not to answer [Go to question 51]

1. . [Female] Have you ever consumed 8 or more alcoholic drinks in one sitting?

- No
- Yes, but not within the last 30 days
- Yes, within the past 30 days
- Prefer not to answer

1. . Have you ever used any kind of nicotine or tobacco product?

- Yes [Go to question 52]
- No [Go to question 53]
- Prefer not to answer [Go to question 53]

1. Which nicotine or tobacco products have you used (check all that apply)?

|  | Tried, but not in past 30 days | Used in the last 30 days | Never |
| --- | --- | --- | --- |
| Cigarette |  |  |  |
| Vaping /E-cigarettes |  |  |  |
| Cigarillo/cigar |  |  |  |
| Hookah |  |  |  |
| Smokeless tobacco product (e.g., chew, snus |  |  |  |
| Nicotine replacement product (e.g., patch, gum) |  |  |  |

1. Have you ever used marijuana or hashish?

- Yes, within the last 30 days
- Yes, but not within the last 30 days
- No
- Prefer not to answer

1. Have you ever used any of the following: (check all the apply)

- Prescription stimulants for NON-medical purposes (e.g., Ritalin, Adderall)
- Prescription painkillers for NON-medical purposes (e.g., oxycontin, Vicodin)
- Other prescription drugs for NON-medical purposes (e.g., Xanax)
- Psychedelics (e.g., LSD, Ecstasy, Molly, mushrooms)
- Any other illicit drug (e.g., cocaine, meth)
- I have NOT used any of the above substances
- Prefer not to answer

1. Which of the following have been a challenge for you since the transition to remote learning? (Check all that apply.)
   - Access to reliable internet service
   - Access to reliable communication software/tools (e.g., Zoom, MS Teams, Google)
   - Access to a reliable digital device (e.g., laptop, tablet, mobile device)
   - Access to specialized software (e.g., Adobe products, statistical packages)
   - Troubleshooting technical issues
   - Distractions (e.g., lack of childcare, lack of quiet work/study space)
   - Other [specify]
   - I have had NO challenges transition to remote learning
   - Prefer not to reply
